# 4-(3-Nitrophenyl)thiazol-2-ylhydrazone derivatives as antioxidants and selective hMAO-B inhibitors: synthesis, biological activity and computational analysis

**DOI:** 10.1080/14756366.2019.1571272

**Published:** 2019-02-06

**Authors:** Daniela Secci, Simone Carradori, Anél Petzer, Paolo Guglielmi, Melissa D’Ascenzio, Paola Chimenti, Donatella Bagetta, Stefano Alcaro, Gokhan Zengin, Jacobus P. Petzer, Francesco Ortuso

**Affiliations:** a Dipartimento di Chimica e Tecnologie del Farmaco, Sapienza University of Rome, Rome, Italy;; b Department of Pharmacy, “G. D’Annunzio” University of Chieti-Pescara, Chieti, Italy;; c Pharmaceutical Chemistry, School of Pharmacy, and Centre of Excellence for Pharmaceutical Sciences, North-West University, Potchefstroom, South Africa;; d Dipartimento di Scienze della Salute, “Magna Graecia” University of Catanzaro, Catanzaro, Italy;; e Department of Biology, Science Faculty, Selcuk University, Konya, Turkey

**Keywords:** (Thiazol-2-yl)hydrazone derivatives, Alzheimer’s disease, Parkinson’s disease, selective, monoamine oxidase, inhibitor, antioxidants, molecular modelling

## Abstract

A new series of 4-(3-nitrophenyl)thiazol-2-ylhydrazone derivatives were designed, synthesised, and evaluated to assess their inhibitory effect on the human monoamine oxidase (hMAO) A and B isoforms. Different (un)substituted (hetero)aromatic substituents were linked to *N*1 of the hydrazone in order to establish robust structure–activity relationships. The results of the biological testing demonstrated that the presence of the hydrazothiazole nucleus bearing at C4 a phenyl ring functionalised at the *meta* position with a nitro group represents an important pharmacophoric feature to obtain selective and reversible human MAO-B inhibition for the treatment of neurodegenerative disorders. In addition, the most potent and selective MAO-B inhibitors were evaluated *in silico* as potential cholinesterase (AChE/BuChE) inhibitors and *in vitro* for antioxidant activities. The results obtained from molecular modelling studies provided insight into the multiple interactions and structural requirements for the reported MAO inhibitory properties.

## 1. Introduction

Neurodegenerative disorders (NDDs) are primarily characterised by an extensive loss of neurons in specific areas of the brain underlying a progressive decline in motor and cognitive functions. The patients suffering from NDDs share a large plethora of pathogenic mechanisms and symptomatology^1^. To overcome such multifactorial diseases, an effective approach should consider molecules able to modulate different pathways. These scaffolds must be chosen among those recognised to interact pleiotropically with important and crucial systems such as monoamine oxidase (MAO-A and MAO-B), cholinesterases [acetylcholinesterase (AChE) and butyrylcholinesterase (BuChE)], and ROS producers. The same molecule, endowed with a multi-target activity and characterised chemically and physically, could represent an innovative advance for the treatment of complex NDDs^2^. Among NDDs, Parkinson’s disease (PD) is of much interest to researchers involved in the design and synthesis of multi-target-direct ligands in the last few years^3^^,^^4^. Among the several proposed scaffolds, we recently explored thiazolylhydrazones as dual-target-directed agents which acts at both MAO-B and AChE, while also possessing antioxidative effects^5^^,^^6^. These compounds displayed very potent inhibitory activity and selectivity against the human monoamine oxidase (hMAO)-B isoform and discrete AChE inhibition and ROS scavenging effects.

Human MAOs are of great medicinal importance due to their unique role in modulating the function/activity of specific neurotransmitters (i.e. dopamine) in various pathogenic cental nervous system (CNS) conditions (PD, mood disorders, anxiety and depression, migraine, aggressive behaviour)^7–13^. Particularly, the expression of the MAO-B isoform in human brain increases with age and has been linked to neurodegeneration, a process which may be attributed to glial cell proliferation (where it is mainly localised) as a result of neuronal loss in specific CNS regions. An excessive dopamine turnover catalyzed by MAO-B with consequent hydrogen peroxide-mediated production of ROS and reactive final products (aldehydes and ammonia) further links MAO-B to neurodegeneration^14^.

Selective MAO-B inhibitors could thus restore the dopamine concentration in the basal ganglia and limit ROS-induced neurotoxicity as well-recognised for the clinically used drugs in this class (selegiline, rasagiline, safinamide)^15^^,^^16^

Previous studies have also shown the correlation between MAO-B and Alzheimer’s disease (AD) due to (i) the increase of MAO-B activity in brain and platelets in AD patients, (ii) the MAO-B specific ligand 11C-deuterium-l-deprenyl showed enhanced binding in presymptomatic familial AD patients, and (iii) AD patients are characterised by enhanced astrocytosis. Moreover, MAO-B was reported to be associated with γ-secretase in the regulation of intraneuronal Aβ levels especially in pyramidal neurons as well as glia cells in the frontal cortex and hippocampus^17^. However, the main pathogenic feature linked with the progression of AD is the weakening of the cholinergic system in the brain and inhibitors of AChE and BuChE are approved as a therapeutic strategy to limit the symptoms and progression of AD. The role of BuChE is not completely known yet.

Recently, we designed and synthesised multi-target inhibitors based on thiazol-2-ylhydrazone scaffold with selective MAO-B inhibitory potency for further structural tuning^18–25^. Pursuing our efforts in the discovery and development of new thiazolylhydrazones effective against NDDs, we extended our study by evaluating derivatives **1–37**, in order to evaluate their hMAOs inhibition profile, chelating properties, antioxidant activity as well as *in silico* AChE and BuChE inhibition properties. These activities determine the efficacy of these compounds to work as multi-target drugs.

## 2. Chemistry

**Scheme 1.**
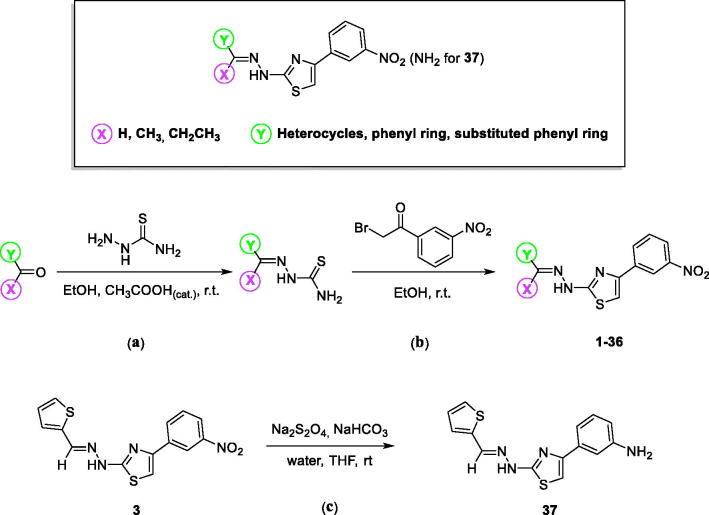
General structure showing the chemical modification made and synthesis of 4-(3-nitrophenyl)thiazol-2-ylhydrazone derivatives **1–36** and 4–(3-aminophenyl)thiazol-2-ylhydrazone derivative **37**.

4–(3-Nitrophenyl)thiazol-2-ylhydrazone derivatives (**1–36**) were synthesised in high yields as reported in our previous communications (Scheme 1)^26^. The appropriate carbonyl compound was reacted with thiosemicarbazide in ethanol at room temperature and in presence of acetic acid as the catalyst (Scheme 1, **a**). The Hantzsch reaction between the resulting thiosemicarbazone and 2-bromo-3′-nitroacetophenone in the same conditions of solvent and temperature gave the corresponding 4–(3-nitrophenyl)thiazol-2-ylhydrazone derivatives (Scheme 1, **b**). For the synthesis of the thiophene-containing 4-(3-amino)thiazol-2-ylhydrazone derivative (**37**), reduction of the nitro group was performed using sodium dithionite previously solubilised in a basic aqueous solution and added dropwise to a stirring suspension of compound **3** in tetrahydrofuran at room temperature (Scheme 1, **c**). All the synthesised products were washed with petroleum ether and diethyl ether and purified by chromatography using silica gel as stationary phase and the appropriate mixtures of ethyl acetate and petroleum ether as mobile phase. Characterisation and purity assessment of the synthesised compounds were carried out by melting point determination, spectroscopic methods (IR, ^1^H and ^13^C NMR) and elemental analysis.

## 3. Biological characterisation

Compounds **1–37** were evaluated *in vitro* as potential inhibitors of the two human recombinant isoforms of monoamine oxidase (hMAO-A and hMAO-B). For a selected compound, the reversibility/irreversibility of MAO inhibition and mechanism of inhibition (e.g. competitive) were evaluated. Furthermore, with the aim to explore the multi-target profile of these derivatives, we performed tests to determine chelating properties, antioxidant activity as well as the AChE and BuChE inhibition profile.

## 4. Experimental protocols

Starting materials and reagents used in the synthetic procedures were obtained from commercial suppliers and were used without further purification. Solvents were freshly distilled before use whenever required. All melting points were measured on a Stuart^®^ melting point apparatus SMP1, and are uncorrected. IR spectra were measured with a PerkinElmer Spectrum 100 FT-IR spectrophotometer equipped with universal total reflectance (ATR) accessory with absorption frequencies expressed in reciprocal centimetres. ^1^H and ^13^ C NMR spectra were recorded at 400 MHz on a Bruker spectrometer using CDCl_3_ and DMSO-d_6_ as the solvents at room temperature. The samples were analyzed at a final concentration of 30 mg/mL. Chemical shifts are expressed as *δ* units (parts per millions) relative to the solvent signal. Coupling constants *J* are valued in Hertz (Hz). The processing and analyses of the NMR data were carried out with MestreNova. Elemental analyses for C, H, and N were recorded on a Perkin-Elmer 240 B microanalyzer obtaining analytical results within ± 0.4% of the theoretical values for all compounds. All reactions were monitored by thin layer chromatography (TLC) performed on 0.2 mm thick silica gel-aluminium backed plates (60 F_254_, Merck). Preparative flash column chromatography was carried out on silica gel (230–400 mesh, G60 Merck). All compounds were recrystallised from ethanol. The yields shown are not optimised. Organic solutions were dried over anhydrous sodium sulphate. Evaporation of the solvent after reaction was carried out on a rotary evaporator (Buchi R-210, Milan, Italy).

### 4.1. General synthetic procedure for the nitro compounds 1–36 and amino compound 37

To a stirring solution of the appropriate carbonyl compound (1.0 eq.) in ethanol (50 ml), thiosemicarbazide (1.0 eq.) and acetic acid as the catalyst were added. The reaction was monitored by TLC up to completion, usually reached in 24–72 h. The obtained suspension was filtered, and the solid washed twice with petroleum ether (20 ml) and diethyl ether (20 ml). The thiosemicarbazone (1.0 eq.), thus synthesised, was reacted with 2-bromo-3′-nitroacetophenone previously dissolved in ethanol (50 ml), and the reaction was magnetically stirred at room temperature until completion as monitored by TLC. The resulting 4–(3-nitrophenyl)thiazol-2-ylhydrazone derivative was collected by filtration, washed with petroleum ether (20 ml) and diethyl ether (20 ml), and purified by column chromatography using ethyl acetate:petroleum ether as mobile phase, to give compounds **1–37** in high yields and purity. With respect to the synthetic approach of compound **36** that is the product of the dimerisation of the parent compound **31**, two equivalents of thiosemicarbazide were used in the first step (Scheme 1, **a**) and two equivalents of 2-bromo-3′-nitroacetophenone were used in the second step (Scheme 1, **b**). To obtain the thiophene containing 4-(3-amino)thiazol-2-ylhydrazone derivative (**37**), sodium dithionite (5.5 eq) was dissolved in a basic solution of water (30 ml) and sodium bicarbonate (5.5 eq.). The resulting solution was added dropwise to a stirring suspension of nitro compound **3** (1.0 eq.) in tetrahydrofuran (50 ml) at room temperature and the reaction was stirred for 2 h (up to completion by TLC). The tetrahydrofuran was evaporated *in vacuo*, the solid that precipitated from the aqueous phase was collected by filtration, washed with water (20 ml) and petroleum ether (20 ml), and dried to give the desired amino derivative **37**. In order to confirm structure and purity of the synthesised test compounds, characterisation was performed using IR, ^1^H NMR and ^13^C NMR along with elemental analysis. Melting points were recorded as mp range. The synthesised compounds exist in theory as a mixture of *E* and *Z* isomers. However, the chemical-physical data are characteristic for the most predominant and thermodynamically stable *E* geometric isomer because of the up-shielding observed in the ^1^H NMR spectra for the NH signal and for the absence of any *Z*-stabilising functional groups. The *Z* isomer is also undesirable due to steric interaction between the aryl group and the NH moiety^27^. In general, the IR spectrum (neat) for derivatives **1**–**37** showed stretching absorption bands at approximately 3310 cm^−1^ for NH, at 3040 cm^−1^due to the stretching of C_sp2_-H, at 1640 cm^−1^ for the C=N and at 1585 and 1445 cm^−1^ for C = C.

#### 4.1.1. 1-(Furan-2-ylmethylene)-2-(4-(3-nitrophenyl)thiazol-2-yl)hydrazine (1)

Green powder, mp 213–215 °C, 71% yield; ^1^H NMR (400 MHz, DMSO-d_6_): *δ* 6.60–6.61 (m, 1H, furan), 6.82–6.83 (m, 1H, furan), 7.64 (s, 1H, C_5_H-thiazole), 7.65–7.70 (m, 1H, furan), 7.79–7.81 (m, 1H, Ar), 7.93 (s, 1H, Ar), 8.14–8.16 (m, 1H, Ar), 8.29–8.31 (m, 1H, Ar), 8.65 (s, 1H, =CH), 12.17 (bs, 1H, NH, D_2_O exch.). Anal. Calcd for C_14_H_10_N_4_O_3_S: C, 53.50; H, 3.21; N, 17.82. Found: C, 53.26; H, 3.01; N, 17.99.

#### 4.1.2. 1-(1-(Furan-2-yl)ethylidene)-2-(4-(3-nitrophenyl)thiazol-2-yl)hydrazine (2)

Yellow powder, mp 230–234 °C, 68% yield; ^1^H NMR (400 MHz, DMSO-d_6_): *δ* 2.28 (s, 3H, CH_3_), 7.38–7.47 (m, 2H, furan), 7.64 (s, 1H, C_5_H-thiazole), 7.66–7.75 (m, 1H, furan), 7.87–7.89 (m, 1H, Ar), 8.15–8.18 (m, 1H, Ar), 8.32–8.35 (m, 1H, Ar), 8.74 (s, 1H, Ar), 11.52 (bs, 1H, NH, D_2_O exch.). Anal. Calcd for C_14_H_10_N_4_O_2_S_2_: C, 54.87; H, 3.68; N, 17.06. Found: C, 55.06; H, 3.51; N, 17.22.

#### 4.1.3. 1-(4-(3-Nitrophenyl)thiazol-2-yl)-2-(thiophen-2-ylmethylene)hydrazine (3)

Yellow powder, mp 187–189 °C, 70% yield; ^1^H NMR (400 MHz, DMSO-d_6_): *δ* 7.09–7.11 (m, 1H, thiophene), 7.37–7.38 (m, 1H, thiophene), 7.58–7.60 (m, 1H, thiophene), 7.64 (s, 1H, C_5_H-thiazole), 7.68–7.72 (t, 1H, Ar), 8.13–8.15 (m, 1H, Ar), 8.23 (s, 1H, Ar), 8.28–8.30 (m, 1H, Ar), 8.66 (s, 1H, =CH), 12.19 (bs, 1H, NH, D_2_O exch.). ^13^C NMR (101 MHz, DMSO-d_6_): *δ* 106.9 (Ar-C5, thiazole), 120.4 (Ar), 122.5 (Ar), 129.8 (Ar, thiophene), 130.7 (Ar), 132.0 (Ar), 134.0 (Ar, thiophene), 136.6 (Ar), 137.5 (Ar, thiophene), 139.5 (Ar, thiophene), 145.9 (C=N), 148.2 (Ar-NO_2_), 148.8 (Ar-C4, thiazole), 168.7 (C=N, thiazole). Anal. Calcd for C_14_H_10_N_4_O_2_S_2_: C, 50.90; H, 3.05; N, 16.96. Found: C, 51.13; H, 2.88; N, 17.20.

#### 4.1.4. 1-(4-(3-Nitrophenyl)thiazol-2-yl)-2-(1-(thiophen-2-yl)ethylidene)hydrazine (4)

Yellow powder, mp 270–272 °C, 74% yield; ^1^H NMR (400 MHz, DMSO-d_6_): *δ* 2.34 (s, 3H, CH_3_), 7.05–7.07 (m, 1H, thiophene), 7.35–7.41 (m, 1H, thiophene), 7.44–7.50 (m, 1H, thiophene), 7.65 (s, 1H, C_5_H-thiazole), 7.69–7.71 (m, 1H, Ar), 8.11–8.14 (m, 1H, Ar), 8.31–8.35 (m, 1H, Ar), 8.70 (s, 1H, Ar), 11.41 (bs, 1H, NH, D_2_O exch.). ^13^C NMR (101 MHz, DMSO-d_6_): *δ* 15.0 (CH_3_), 107.4 (Ar-C5, thiazole), 120.4 (Ar), 122.5 (Ar), 127.1 (Ar, thiophene), 128.1 (Ar, thiophene), 128.4 (Ar, thiophene), 130.7 (Ar), 132.0 (Ar), 136.7 (Ar), 143.7 (Ar, thiophene), 144.6 (C=N), 148.5 (Ar-NO_2_), 148.8 (Ar-C4, thiazole), 168.7 (C=N, thiazole). Anal. Calcd for C_15_H_12_N_4_O_2_S_2_: C, 52.31; H, 3.51; N, 16.27. Found: C, 52.05; H, 3.78; N, 16.43.

#### 4.1.5. 1-(4-(3-Nitrophenyl)thiazol-2-yl)-2-(1-(thiophen-3-yl)ethylidene)hydrazine (5)

Yellow powder, mp 183–185 °C, 85% yield; ^1^H NMR (400 MHz, CDCl_3_): *δ* 2.39 (s, 3H, CH_3_), 7.07 (s, 1H, C_5_H-thiazole), 7.36–7.38 (m, 1H, Ar), 7.57 (s, 1H, thiophene), 7.61–7.65 (m, 2H, thiophene), 8.16–8.22 (m, 2H, Ar), 8.66 (s, 1H, Ar), 9.49 (bs, 1H, NH, D_2_O exch.). ^13^C NMR (101 MHz, DMSO-d_6_): *δ* 15.2 (CH_3_), 107.2 (Ar-C5, thiazole), 120.4 (Ar), 122.4 (Ar), 124.7 (Ar, thiophene), 125.7 (Ar, thiophene), 127.3 (Ar, thiophene), 130.7 (Ar), 132.0 (Ar), 136.7 (Ar), 141.7 (Ar, thiophene), 144.6 (C=N), 148.5 (Ar-NO_2_), 148.8 (Ar-C4, thiazole), 168.7 (C=N, thiazole). Anal. Calcd for C_15_H_12_N_4_O_2_S_2_: C, 52.31; H, 3.51; N, 16.27. Found: C, 52.60; H, 3.27; N, 16.02.

#### 4.1.6. 1-(1-(5-Chlorothiophen-2-yl)ethylidene)-2-(4-(3-nitrophenyl)thiazol-2-yl)hydrazine (6)

Orange powder, mp 227–229 °C, 81% yield; ^1^H NMR (400 MHz, DMSO-d_6_): *δ* 2.29 (s, 3H, CH_3_), 7.09 (s, 1H, C_5_H-thiazole), 7.26–7.29 (m, 1H, Ar), 7.67–7.73 (m, 2H, thiophene), 8.14–8.32 (m, 2H, Ar), 8.70 (s, 1H, Ar), 11.58 (bs, 1H, NH, D_2_O exch.). Anal. Calcd for C_15_H_11_ClN_4_O_2_S_2_: C, 47.55; H, 2.93; N, 14.79. Found: C, 47.71; H, 3.17; N, 14.53.

#### 4.1.7. 1-Benzylidene-2-(4-(3-nitrophenyl)thiazol-2-yl)hydrazine (7)

Yellow powder, mp 193–195 °C, 76% yield; ^1^H NMR (400 MHz, DMSO-d_6_): *δ* 7.38–7.47 (m, 3H, Ar), 7.67–7.74 (m, 3H, Ar), 8.06 (s, 1H, C_5_H-thiazole), 8.15–8.17 (m, 1H, Ar), 8.30–8.32 (m, 1H, Ar), 8.41–8.43 (m, 1H, Ar), 8.69 (s, 1H, =CH), 12.31 (bs, 1H, NH, D_2_O exch.). Anal. Calcd for C_16_H_12_N_4_O_2_S: C, 59.25; H, 3.73; N, 17.27. Found: C, 58.99; H, 3.98; N, 17.02.

#### 4.1.8. 1-(2-Nitrobenzylidene)-2-(4-(3-nitrophenyl)thiazol-2-yl)hydrazine (8)

Orange powder, mp 228–230 °C, 79% yield; ^1^H NMR (400 MHz, DMSO-d_6_): *δ* 7.63–7.65 (m, 1H, Ar), 7.73–7.74 (m, 2H, Ar), 7.79–7.82 (m, 1H, Ar), 8.04–8.05 (m, 1H, Ar), 8.06 (s, 1H, C_5_H-thiazole), 8.16–8.18 (m, 1H, Ar), 8.31–8.33 (m, 1H, Ar), 8.45–8.47 (m, 1H, Ar), 8.69 (s, 1H, =CH), 12.68 (bs, 1H, NH, D_2_O exch.). ^13^C NMR (101 MHz, DMSO-d_6_): *δ* 107.6 (Ar-C5, thiazole), 120.4 (Ar), 122.6 (Ar), 125.2 (Ar), 128.0 (Ar), 128.9 (Ar), 130.3 (Ar), 130.7 (Ar), 132.1 (Ar), 134.0 (Ar), 136.5 (Ar), 137.2 (Ar), 137.6 (C=N), 147.9 (Ar-NO_2_), 148.8 (Ar-C4, thiazole), 168.6 (C=N, thiazole). Anal. Calcd for C_16_H_11_N_5_O_4_S: C, 52.03; H, 3.00; N, 18.96. Found: C, 52.27; H, 2.82; N, 19.12.

#### 4.1.9. 1-(3-Nitrobenzylidene)-2-(4-(3-nitrophenyl)thiazol-2-yl)hydrazine (9)

Yellow powder, mp 222–224 °C, 81% yield; ^1^H NMR (400 MHz, DMSO-d_6_): *δ* 7.67–7.73 (m, 2H, Ar), 8.15 (s, 1H, C_5_H-thiazole), 8.17–8.31 (m, 6H, Ar), 8.66 (s, 1H, =CH), 11.62 (bs, 1H, NH, D_2_O exch.). Anal. Calcd for C_16_H_11_N_5_O_4_S: C, 52.03; H, 3.00; N, 18.96. Found: C, 52.21; H, 3.24; N, 18.74.

#### 4.1.10. 1-(4-Nitrobenzylidene)-2-(4-(3-nitrophenyl)thiazol-2-yl)hydrazine (10)

Characterisation data were in agreement with those reported in the literature^28^.

#### 4.1.11. 1-(4-(3-Nitrophenyl)thiazol-2-yl)-2-(1-phenylethylidene)hydrazine (11)

Yellow powder, mp 261–263 °C, 86% yield; ^1^H NMR (400 MHz, DMSO-d_6_): *δ* 2.34 (s, 3H, CH_3_), 7.38–7.45 (m, 3H, Ar), 7.67 (s, 1H, C_5_H-thiazole), 7.69–7.73 (m, 1H, Ar), 7.77–7.80 (m, 2H, Ar), 8.13–8.16 (m, 1H, Ar), 8.31–8.33 (m, 1H, Ar), 8.72–8.73 (m, 1H, Ar), 11.36 (bs, 1H, NH, D_2_O exch.). ^13^C NMR (101 MHz, DMSO-d_6_): *δ* 14.6 (CH_3_), 107.3 (Ar-C5, thiazole), 120.5 (Ar), 122.4 (Ar), 126.2 (2 × Ar), 128.9 (2 × Ar), 129.3 (Ar), 130.7 (Ar), 132.0 (Ar), 136.8 (Ar), 138.3 (Ar), 147.4 (C=N), 148.6 (Ar-NO_2_), 148.8 (Ar-C4, thiazole), 170.6 (C=N, thiazole). Anal. Calcd for C_17_H_14_N_4_O_2_S: C, 60.34; H, 4.17; N, 16.56. Found: C, 60.12; H, 4.32; N, 16.79.

#### 4.1.12. 2-(4-(3-Nitrophenyl)thiazol-2-yl)-1-(1-phenylpropylidene)hydrazine (12)

Yellow powder, mp 215–217 °C, 85% yield; ^1^H NMR (400 MHz, DMSO-d_6_): *δ* 1.08–1.12 (t, 3H, CH_3_), 2.86–2.91 (m, 2H, CH_2_), 7.38–7.46 (m, 3H, Ar), 7.69 (s, 1H, C_5_H-thiazole), 7.70–7.74 (m, 1H, Ar), 7.79–7.81 (m, 2H, Ar), 8.15–8.17 (d, *J* = 8.0 Hz, 1H, Ar), 8.32–8.34 (d, *J* = 8.0 Hz, 1H, Ar), 8.74 (s, 1H, Ar), 11.55 (bs, 1H, NH, D_2_O exch.). Anal. Calcd for C_18_H_16_N_4_O_2_S: C, 61.35; H, 4.58; N, 15.90. Found: C, 61.20; H, 4.32; N, 16.13.

#### 4.1.13. 1-(1-(2-Nitrophenyl)ethylidene)-2-(4-(3-nitrophenyl)thiazol-2-yl)hydrazine (13)

Orange powder, mp 195–200 °C, 87% yield; ^1^H NMR (400 MHz, DMSO-d_6_): *δ* 2.34 (s, 3H, CH_3_), 7.61–7.65 (m, 2H, 1H Ar + 1H C_5_H-thiazole), 7.70–7.78 (m, 3H, Ar), 7.91–7.93 (m, 1H, Ar), 8.15–8.17 (m, 1H, Ar), 8.30–8.31 (m, 1H, Ar), 8.71 (s, 1H, Ar), 11.51 (bs, 1H, NH, D_2_O exch.). Anal. Calcd for C_17_H_13_N_5_O_4_S: C, 53.26; H, 3.42; N, 18.27. Found: C, 53.11; H, 3.25; N, 18.54.

#### 4.1.14. 1-(1-(3-Nitrophenyl)ethylidene)-2-(4-(3-nitrophenyl)thiazol-2-yl)hydrazine (14)

Orange powder, mp 214–216 °C, 85% yield; ^1^H NMR (400 MHz, CDCl_3_): *δ* 2.40 (s, 3H, CH_3_), 7.14 (s, 1H, C_5_H-thiazole), 7.61–7.63 (m, 2H, Ar), 8.15–8.26 (m, 4H, Ar), 8.63 (s, 1H, Ar), 8.70 (s, 1H, Ar), 8.98 (bs, 1H, NH, D_2_O exch.). Anal. Calcd for C_17_H_13_N_5_O_4_S: C, 53.26; H, 3.42; N, 18.27. Found: C, 53.55; H, 3.70; N, 18.09.

#### 4.1.15. 1-(1-(4-Nitrophenyl)ethylidene)-2-(4-(3-nitrophenyl)thiazol-2-yl)hydrazine (15)

Orange powder, mp 258–260 °C, 80% yield; ^1^H NMR (400 MHz, DMSO-d_6_): *δ* 2.41 (s, 3H, CH_3_), 7.71–7.75 (m, 2H, 1H Ar + 1H C_5_H-thiazole), 8.02–8.05 (d, *J* = 8.8 Hz, 2H, Ar), 8.16–8.18 (m, 1H, Ar), 8.28–8.30 (d, *J* = 8.8 Hz, 2H, Ar), 8.32–8.35 (m, 1H, Ar), 8.74 (s, 1H, Ar), 11.73 (bs, 1H, NH, D_2_O exch.). Anal. Calcd for C_17_H_13_N_5_O_4_S: C, 53.26; H, 3.42; N,18.27. Found: C, 53.01; H, 3.18; N, 18.43.

#### 4.1.16. 1-(4-(3-Nitrophenyl)thiazol-2-yl)-2-(pyridin-2-ylmethylene)hydrazine (16)

Characterisation data were in agreement with those reported in the literature^29^.

#### 4.1.17. 1-(4-(3-Nitrophenyl)thiazol-2-yl)-2-(pyridin-3-ylmethylene)hydrazine (17)

Characterisation data were in agreement with those reported in the literature^30^.

#### 4.1.18. 1-(4-(3-Nitrophenyl)thiazol-2-yl)-2-(pyridin-4-ylmethylene)hydrazine (18)

Characterisation data were in agreement with those reported in the literature^31^.

#### 4.1.19. 1-(4-(3-Nitrophenyl)thiazol-2-yl)-2-(1-(pyridin-2-yl)ethylidene)hydrazine (19)

Characterisation data were in agreement with those reported in the literature^30^.

#### 4.1.20. 1-(4-(3-Nitrophenyl)thiazol-2-yl)-2-(1-(pyridin-3-yl)ethylidene)hydrazine (20)

Characterisation data were in agreement with those reported in the literature^30^.

#### 4.1.21. 1-(4-(3-Nitrophenyl)thiazol-2-yl)-2-(1-(pyridin-4-yl)ethylidene)hydrazine (21)

Characterisation data were in agreement with those reported in the literature^30^.

#### 4.1.22. 1-(4-(3-Nitrophenyl)thiazol-2-yl)-2-(1-(pyrazin-2-yl)ethylidene)hydrazine (22)

Yellow powder, mp 243–245 °C, 85% yield; ^1^H NMR (400 MHz, DMSO-d_6_): *δ* 2.41 (s, 1H, CH_3_), 7.71–7.75 (m, 1H, Ar), 7.77 (s, 1H, C_5_H-thiazole), 8.16–8.18 (m, 1H, Ar), 8.33–8.35 (m, 1H, Ar), 8.59–8.60 (m, 1H, Ar), 8.63–8.64 (m, 1H, Ar), 8.74–8.75 (m, 1H, Ar), 9.22–9.23 (m, 1H, Ar), 11.82 (bs, 1H, NH, D_2_O exch.). Anal. Calcd for C_15_H_12_N_6_O_2_S: C, 52.93; H, 3.55; N, 24.69. Found: C, 53.20; H, 3.74; N, 24.84.

#### 4.1.23. 1-((1H-Indol-3-yl)methylene)-2-(4-(3-nitrophenyl)thiazol-2-yl)hydrazine (23)

Yellow powder, mp 253–255 °C, 83% yield; ^1^H NMR (400 MHz, DMSO-d_6_): *δ* 7.20–7.25 (m, 2H, Ar), 7.45–7.47 (m, 1H, Ar), 7.62 (s, 1H, C_5_H-thiazole), 7.70–7.74 (m, 1H, Ar), 7.79–7.80 (m, 1H, Ar), 8.15–8.17 (m, 1H, Ar), 8.23–8.25 (m, 1H, Ar), 8.29–8.33 (m, 2H, Ar), 8.70 (s, 1H, =CH), 11.55 (bs, 1H, NH-indole, D_2_O exch.), 11.98 (bs, 1H, NH, D_2_O exch.). ^13^C NMR (101 MHz, DMSO-d_6_): *δ* 106.0 (Ar-C5, thiazole), 112.0 (Ar, indole), 112.4 (Ar, indole), 120.4 (Ar), 121.0 (Ar, indole), 122.1 (Ar), 122.5 (Ar, indole), 123.1 (Ar, indole), 124.5 (Ar, indole), 130.3 (Ar, indole), 130.7 (Ar), 132.1 (Ar), 136.6 (Ar), 137.6 (Ar, indole), 140.6 (C=N), 148.0 (Ar-NO_2_), 148.7 (Ar-C4, thiazole), 168.7 (C=N, thiazole). Anal. Calcd for C_18_H_13_N_5_O_2_S: C, 59.49; H, 3.61; N, 19.27. Found: C, 59.65; H, 3.39; N, 19.49.

#### 4.1.24. 1-(1-(1H-Indol-3-yl)ethylidene)-2-(4-(3-nitrophenyl)thiazol-2-yl)hydrazine (24)

Green powder, mp 209–211 °C, 87% yield; ^1^H NMR (400 MHz, DMSO-d_6_): *δ* 2.36 (s, 3H, CH_3_), 7.25–7.28 (m, 2H, 1H C_5_H-thiazole + 1H Ar), 7.43–7.47 (m, 1H, Ar), 7.63–7.90 (m, 3H, Ar), 8.12–8.21 (m, 1H, Ar), 8.31–8.50 (m, 2H, Ar), 8.78 (s, 1H, Ar), 11.08 (bs, 1H, NH-indole, D_2_O exch.), 11.48 (bs, 1H, NH, D_2_O exch.). Anal. Calcd for C_19_H_15_N_5_O_2_S: C, 60.46; H, 4.01; N, 18.56. Found: C, 60.70; H, 3.83; N, 18.37.

#### 4.1.25. 1-(Benzo[d][1,3]dioxol-5-ylmethylene)-2-(4-(3-nitrophenyl)thiazol-2-yl)hydrazine (25)

Yellow powder, mp 255–256 °C, 84% yield; ^1^H NMR (400 MHz, DMSO-d_6_): *δ* 6.08 (s, 2H, OCH_2_O), 6.96–6.98 (m, 1H, Ar), 7.11–7.13 (m, 1H, Ar), 7.23 (s, 1H, Ar), 7.63 (s, 1H, C_5_H-thiazole), 7.68–7.72 (m, 1H, Ar), 7.96 (s, 1H, Ar), 8.13–8.15 (m, 1H, Ar), 8.28–8.30 (m, 1H, Ar), 8.66 (s, 1H, =CH), 12.17 (bs, 1H, NH, D_2_O exch.). ^13^C NMR (101 MHz, DMSO-d_6_): *δ* 101.9 (OCH_2_O, benzodioxole), 105.1 (Ar, benzodioxole), 106.7 (Ar-C5, thiazole), 109.0 (Ar, benzodioxole), 120.4 (Ar), 122.4 (Ar), 122.7 (Ar, benzodioxole), 129.2 (Ar, benzodioxole), 130.7 (Ar), 132.0 (Ar), 136.7 (Ar), 142.0 (C=N), 148.4 (Ar-NO_2_), 148.6 (Ar, benzodioxole), 148.7 (Ar-C4, thiazole), 149.0 (Ar, benzodioxole), 169.1 (C=N, thiazole). Anal. Calcd for C_17_H_12_N_4_O_4_S: C, 55.43; H, 3.28; N, 15.21. Found: C, 55.22; H, 3.04; N, 15.39.

#### 4.1.26. 1-(1-(Benzofuran-2-yl)ethylidene)-2-(4-(3-nitrophenyl)thiazol-2-yl)hydrazine (26)

Pink powder, mp 228–230 °C, 81% yield; ^1^H NMR (400 MHz, DMSO-d_6_): *δ* 2.37 (s, 3H, CH_3_), 7.25–7.29 (m, 1H, Ar), 7.31(s, 1H, C_5_H-thiazole), 7.34–7.37 (m, 1H, Ar), 7.61–7.63 (m, 4H, Ar) 8.14–8.16 (m, 1H, Ar), 8.32–8.34 (m, 1H, Ar), 8.73 (s, 1H, Ar), 11.61 (bs, 1H, NH, D_2_O exch.). ^13^C NMR (101 MHz, DMSO-d_6_): *δ* 14.3 (CH_3_), 106.6 (Ar-C5, thiazole), 107.7 (Ar, benzofuran), 111.7 (Ar, benzofuran), 120.5 (Ar), 122.0 (Ar, benzofuran), 122.5 (Ar), 123.8 (Ar, benzofuran), 125.9 (Ar, benzofuran), 128.6 (Ar, benzofuran), 130.7 (Ar), 132.0 (Ar), 136.7 (Ar), 139.1 (C=N), 148.6 (Ar-NO_2_), 148.8 (Ar-C4, thiazole), 154.0 (Ar, benzofuran), 155.0 (Ar, benzofuran), 169.9 (C=N, thiazole). Anal. Calcd for C_19_H_14_N_4_O_3_S: C, 60.31; H, 3.73; N, 14.81. Found: C, 60.07; H, 3.97; N, 15.00.

#### 4.1.27. 1-(1-(2,3-Dihydrobenzo[b][1,4]dioxin-6-yl)ethylidene)-2-(4-(3-nitrophenyl)thiazol-2-yl)hydrazine (27)

Orange powder, mp 242–244 °C, 70% yield; ^1^H NMR (400 MHz, DMSO-d_6_): *δ* 2.28 (s, 3H, CH_3_), 4.28 (s, 4H, benzodioxine), 6.89–6.91 (m, 1H, Ar), 7.27–7.29 (m, 2H, Ar), 7.66 (s, 1H, C_5_H-thiazole), 7.70–7.74 (t, 1H, Ar), 8.15–8.17 (m, 1H, Ar), 8.31–8.33 (m, 1H, Ar), 8.73 (s, 1H, Ar), 11.28 (bs, 1H, NH, D_2_O exch.). ^13^C NMR (101 MHz, DMSO-d_6_): *δ* 14.5 (CH_3_), 64.5 (CH_2_), 64.7 (CH_2_), 107.2 (Ar-C5, thiazole), 114.9 (Ar, benzodioxine), 117.4 (Ar, benzodioxine), 119.5 (Ar, benzodioxine), 120.4 (Ar), 122.4 (Ar), 130.7 (Ar), 131.6 (Ar, benzodioxine), 132.0 (Ar), 136.7 (Ar), 143.6 (C=N), 144.7 (Ar, benzodioxine), 147.1 (Ar, benzodioxine), 148.5 (Ar-NO_2_), 148.7 (Ar-C4, thiazole), 170.7 (C=N, thiazole). Anal. Calcd for C_19_H_16_N_4_O_4_S: C, 57.57; H, 4.07; N, 14.13. Found: C, 57.80; H, 3.84; N, 58.02.

#### 4.1.28. 1-(Naphthalen-1-ylmethylene)-2-(4-(3-nitrophenyl)thiazol-2-yl)hydrazine (28)

Orange powder, mp 212–214 °C, 72% yield; ^1^H NMR (400 MHz, DMSO-d_6_): *δ* 7.59–7.63 (m, 2H, Ar), 7.67–7.75 (m, 3H, 2H Ar + 1H C_5_H-thiazole), 7.87–7.89 (d, *J* = 8.0 Hz, 1H, Ar), 7.99–8.03 (t, 2H, Ar), 8.16–8.18 (m, 1H, Ar), 8.33–8.35 (d, *J* = 8.0 Hz, 1H, Ar), 8.70–8.71 (m, 2H, 1H Ar + 1H = CH), 8.77–8.79 (d, *J* = 8.0 Hz, 1H, Ar), 12.52 (bs, 1H, NH, D_2_O exch.). Anal. Calcd for C_20_H_14_N_4_O_2_S: C, 64.16; H, 3.77; N, 14.96. Found: C, 63.92; H, 3.90; N, 15.12.

#### 4.1.29. 1-(1-(Naphthalen-1-yl)ethylidene)-2-(4-(3-nitrophenyl)thiazol-2-yl)hydrazine (29)

White powder, mp 226–228 °C, 74% yield; ^1^H NMR (400 MHz, DMSO-d_6_): *δ* 2.33 (s, 3H, CH_3_), 7.54–7.59 (m, 4H, 3H Ar + 1H C_5_H-thiazole), 7.91–8.00 (m, 4H, Ar), 8.26–8.41 (m, 2H, Ar), 8.56–8.58 (m, 1H, Ar), 8.71–8.75 (m, 1H, Ar), 12.14 (bs, 1H, NH, D_2_O exch.). Anal. Calcd for C_21_H_16_N_4_O_2_S: C, 64.93; H, 4.15; N, 14.42. Found: C, 65.10; H, 3.89; N, 14.21.

#### 4.1.30. 1-(1-(Naphthalen-2-yl)ethylidene)-2-(4-(3-nitrophenyl)thiazol-2-yl)hydrazine (30)

Yellow powder, mp 152–154 °C, 77% yield; ^1^H NMR (400 MHz, DMSO-d_6_): *δ* 2.36 (s, 3H, CH_3_), 7.53–7.55 (m, 2H, Ar), 7.70–7.72 (m, 2H, 1H Ar + 1H C_5_H-thiazole), 7.90–7.94 (m, 2H, Ar), 7.96–7.98 (m, 1H, Ar), 8.03–8.05 (m, 1H, Ar), 8.07–8.09 (m, 1H, Ar), 8.22–8.24 (m, 1H, Ar), 8.41–8.43 (m, 1H, Ar), 8.74–8.75 (m, 1H, Ar), 11.54 (bs, 1H, NH, D_2_O exch.). Anal. Calcd for C_21_H_16_N_4_O_2_S: C, 64.93; H, 4.15; N, 14.42. Found: C, 64.74; H, 4.36; N, 14.65.

#### 4.1.31. 1-(1-(4-Acetylphenyl)ethylidene)-2-(4-(3-nitrophenyl)thiazol-2-yl)hydrazine (31)

Yellow powder, mp 205–207 °C, 79% yield; ^1^H NMR (400 MHz, CDCl_3_): *δ* 2.44 (s, 3H, CH_3_), 2.66 (s, 3H, CH_3_), 7.10 (s, 1H, C_5_H-thiazole), 7.64–7.66 (t, 1H, Ar), 7.90–7.92 (d, *J* = 8.4 Hz, 2H, Ar), 8.02–8.04 (d, *J* = 8.0 Hz, 2H, Ar), 8.15–8.17 (d, *J* = 6.8 Hz, 1H, Ar), 8.21–8.23 (d, *J* = 6.8 Hz, 1H, Ar), 8.65 (s, 1H, Ar), 10.15 (bs, 1H, NH, D_2_O exch.). ^13^C NMR (101 MHz, DMSO-d_6_): *δ* 14.3 (CH_3_), 27.2 (CH_3_), 107.7 (Ar-C5, thiazole), 120.5 (Ar), 122.5 (Ar), 126.2 (2 × Ar), 128.8 (2 × Ar), 130.7 (Ar), 132.0 (Ar), 136.7 (Ar), 136.9 (Ar), 142.4 (C=N), 146.0 (Ar-NO_2_), 148.8 (Ar-C4, thiazole), 170.3 (C=N, thiazole), 197.9 (C=O). Anal. Calcd for C_19_H_16_N_4_O_3_S: C, 59.99; H, 4.24; N, 14.73. Found: C, 60.15; H, 4.03; N, 15.00.

#### 4.1.32. 1-(Diphenylmethylene)-2-(4-(3-nitrophenyl)thiazol-2-yl)hydrazine (32)

Yellow powder, mp 166–168 °C, 83% yield; ^1^H NMR (400 MHz, CDCl_3_): *δ* 7.05 (s, 1H, C_5_H-thiazole), 7.39–7.43 (m, 5H, Ar), 7.62–7.67 (m, 6H, Ar), 8.14–8.25 (m, 2H, Ar), 8.58 (s, 1H, Ar), 10.15 (bs, 1H, NH, D_2_O exch.). ^13^C NMR (101 MHz, DMSO-d_6_): *δ* 105.7 (Ar-C5, thiazole), 120.7 (Ar), 122.6 (Ar), 127.4 (2 × Ar), 128.4 (2 × Ar), 128.6 (2 × Ar), 129.7 (Ar), 129.8 (Ar), 130.0 (2 × Ar), 130.2 (Ar), 131.5 (Ar), 131.6 (Ar), 135.3 (C=N), 136.6 (Ar), 147.4 (Ar-NO_2_), 148.7 (Ar-C4, thiazole), 168.8 (C=N, thiazole). Anal. Calcd for C_22_H_16_N_4_O_2_S: C, 65.98; H, 4.03; N, 13.99. Found: C, 66.14; H, 4.21; N, 14.17.

#### 4.1.33. 1-(1-(Coumarin-3-yl)ethylidene)-2-(4-(3-nitrophenyl)thiazol-2-yl)hydrazine (33)

Red powder, mp 183–185 °C, 75% yield; ^1^H NMR (400 MHz, DMSO-d_6_): *δ* 2.26 (s, 3H, CH_3_), 6.60 (s, 1H, C_5_H-thiazole), 6.86–6.87 (m, 1H, Ar), 7.66–7.79 (m, 5H, Ar), 8.15–8.17 (m, 1H, Ar), 8.31–8.33 (m, 1H, Ar), 8.72 (s, 1H, Ar), 11.35 (bs, 1H, NH, D_2_O exch.). ^13^C NMR (101 MHz, DMSO-d_6_): *δ* 14.2 (CH_3_), 107.3 (Ar-C5, thiazole), 110.6 (Ar, coumarin), 112.3 (Ar, coumarin), 120.4 (Ar), 122.4 (Ar), 130.7 (Ar), 132.0 (Ar), 136.7 (Ar), 139.7 (Ar, coumarin), 144.6 (C=N), 148.6 (Ar-NO_2_), 148.8 (Ar-C4, thiazole), 152.2 (C=O, coumarin), 170.2 (C=N, thiazole). Anal. Calcd for C_20_H_14_N_4_O_4_S: C, 59.11; H, 3.47; N, 13.79. Found: C, 58.94; H, 3.24; N, 13.98.

#### 4.1.34. 1-(4-(3-Nitrophenyl)thiazol-2-yl)-2-(1-(phenanthren-3-yl)ethylidene)hydrazine (34)

Pink powder, mp 255–257 °C, 72% yield; ^1^H NMR (400 MHz, DMSO-d_6_): *δ* 1.24 (s, 3H, CH_3_), 7.25 (s, 2H, 1H Ar + 1H C_5_H-thiazole), 7.37–7.48 (m, 5H, Ar), 7.61–7.69 (m, 5H, Ar), 8.01–8.03 (d, *J* = 8.4 Hz, 2H, Ar), 11.40 (bs, 1H, NH, D_2_O exch.). Anal. Calcd for C_25_H_18_N_4_O_2_S: C, 68.48; H, 4.14; N, 12.78. Found: C, 68.30; H, 3.97; N, 12.54.

#### 4.1.35. 1-(1-(Ferrocen-2-yl)ethylidene)-2-(4-(3-nitrophenyl)thiazol-2-yl)hydrazine (35)

Orange powder, mp 156–158 °C, 80% yield; ^1^H NMR (400 MHz, CDCl_3_): *δ* 2.49 (s, 3H, CH_3_), 4.24 (s, 4H, ferrocene), 4.34 (s, 1H, ferrocene), 4.50 (s, 2H, ferrocene), 4.72 (s, 2H, ferrocene), 6.99 (s, 1H, C_5_H-thiazole), 7.79 (s, 1H, Ar), 8.22–8.32 (m, 2H, Ar), 8.58 (s, 1H, Ar), 12.35 (bs, 1H, NH, D_2_O exch.). Anal. Calcd for C_21_H_18_FeN_4_O_2_S: C, 56.51; H, 4.07; N, 12.55. Found: C, 56.73; H, 4.29; N, 12.80.

#### 4.1.36. 1,4-bis(1-(2-(4-(3-nitrophenyl)thiazol-2-yl)hydrazono)ethyl)benzene (36)

Orange powder, mp 283–285 °C, 81% yield; ^1^H NMR (400 MHz, DMSO-d_6_): *δ* 2.37 (s, 6H, 2 × CH_3_), 7.71–7.75 (m, 3H, 1H Ar + 2H 2 × C_5_H-thiazole), 7.86 (bs, 3H, Ar), 7.87–7.88 (m, 1H, Ar), 7.89–7.90 (m, 1H, Ar), 8.16–8.18 (m, 2H, Ar), 8.33–8.35 (m, 2H, Ar), 8.75 (s, 2H, Ar), 11.45 (bs, 2H, 2 × NH, D_2_O exch.). Anal. Calcd for C_28_H_22_N_8_O_4_S_2_: C, 56.18; H, 3.70; N, 18.72. Found: C, 56.01; H, 3.96; N, 18.55.

#### 4.1.37. 1-(4-(3-Aminophenyl)thiazol-2-yl)-2-(1-(thiophen-2-yl)ethylidene)hydrazine (37)

Red powder, mp 165–167 °C, 71% yield; ^1^H NMR (400 MHz, DMSO-d_6_): *δ* 5.15 (bs, 2H, NH_2_, D_2_O exch.), 6.50 (s, 1H, C_5_H-thiazole), 6.99–7.12 (m, 5H, 4H Ar + 1H thiophene), 7.36 (s, 1H, thiophene), 7.58–7.59 (m, 1H, thiophene), 8.21 (s, 1H, CH=), 12.06 (bs, 1H, NH, D_2_O exch.). Anal. Calcd for C_14_H_12_N_4_S_2_: C, 55.97; H, 4.03; N, 18.65. Found: C, 56.15; H, 3.84; N, 18.37.

### 4.2. MAO-A and MAO-B inhibition studies

#### 4.2.1. Determination of IC_50_ values

The procedure for the measurement of IC_50_ values for the inhibition of MAO has been reported^32^. The enzyme reactions were prepared to a volume of 200 µM in potassium phosphate buffer at pH 7.4 (100 mM, made isotonic with KCl) and contained kynuramine (50 μM) and the test inhibitors (0.003–100 µM). Stock solutions of the test inhibitors were prepared in DMSO and added to the reactions to yield 4% DMSO. The enzyme reactions were initiated with the addition of recombinant hMAO-A (0.0075 mg protein/mL) or hMAO-B (0.015 mg protein/mL) and incubated for 20 min at 37 °C. At endpoint, the reactions were terminated with 80 µL sodium hydroxide (2N) and the fluorescence of 4-hydroxyquinoline, the oxidation product of kynuramine, was measured (*λ*_ex_ = 310; *λ*_em_ = 400 nm)^33^. 4-Hydroxyquinoline was quantified with a calibration curve (0.047–1.56 μM) and the rate of 4-hydroxyquinoline formation was fitted to the one site competition model of the Prism 5 software package (GraphPad). From the resulting sigmoidal plots, the IC_50_ values were estimated. All enzyme reactions were carried out in triplicate and IC_50_ values are given as the mean ± standard deviation (SD).

#### 4.2.2. Investigating reversibility of inhibition by dialysis

The procedure for the measurement of IC_50_ values for the inhibition of MAO has been reported^32^. hMAO-B (0.03 mg/mL) and the test compounds (at 4 × IC_50_) were incubated for 15 min at 37 °C to a final volume of 0.8 ml in dialysis buffer, potassium phosphate buffer (100 mM, pH 7.4) containing 5% sucrose. Stock solutions of the test compounds were prepared in DMSO and added to the incubations to yield 4% DMSO. The samples were dialyzed for 24 h at 4 °C in 80 ml of dialysis buffer using Slide-A-Lyzer^®^ dialysis cassettes (Thermo Scientific) with a molecular weight cut-off of 10,000 and a sample volume capacity of 0.5–3 ml. The dialysis buffer was replaced at 3 and 7 h after the start of dialysis. Following dialysis, the dialysis samples were diluted twofold with the addition of kynuramine to yield a kynuramine concentration and inhibitor concentration of 50 µM and 2 × IC_50_, respectively. The reactions (500 µL) were incubated for 20 min at 37 °C and terminated with NaOH (400 μL, 2 N) and water (1000 µL). The fluorescence of 4-hydroxyquinoline in these samples was measured as described for the IC_50_ determination above. As controls, MAO-B was similarly pre-incubated and dialyzed in the absence of inhibitor (negative control) as well as in the presence of the irreversible inhibitor, (R)-(−)-deprenyl (positive control; IC_50_ = 0.079 μM)^34^. Undialysed mixtures of MAO-B and the test inhibitors were maintained at 4 °C for 24 h and diluted and assayed as above. All reactions were carried out in triplicate and the residual enzyme catalytic rates are expressed as mean ± SD.

#### 4.2.3. Lineweaver–Burk plots

To construct Lineweaver–Burk plots, the enzyme reactions were carried out to a volume of 500 µL and the concentration of MAO-B was 0.015 mg protein/mL. All enzyme reactions and activity measurements were carried out as described above for the dialysis experiments. The first plot was constructed in the absence of inhibitor, while the remaining five plots were constructed in the presence of the following concentrations: ¼ × IC_50_, ½ × IC_50_, ¾ × IC_50_, 1 × IC_50_ and 1¼ × IC_50_. For each line, kynuramine was used at concentrations of 15–250 μM. The *K*_i_ value was estimated from a plot of the slopes of the Lineweaver–Burk plots versus inhibitor concentration (*x*-axis intercept equals –*K*_i_).

### 4.3. Antioxidant activity evaluation

#### 4.3.1. Phosphomolybdenum assay (PhosphoMo)

The total antioxidant activity of the compounds was evaluated by phosphomolybdenum method according to Zengin et al.^35^. The sample solution (0.3 ml) was combined with 3 ml of reagent solution (0.6 M sulphuric acid, 28 mM sodium phosphate, and 4 mM ammonium molybdate) and the absorbance was recorded at 695 nm after 90 min incubation at 95 °C. The EC_50_, which is the effective concentration at which the absorbance was 0.5, was calculated for each compound and trolox, as a reference drug.

#### 4.3.2. Radical scavenging activity (DPPH and ABTS)

The radical scavenging effect of the compounds using the the 1,1-diphenyl-2-picrylhydrazyl (DPPH) radical was estimated according to Zengin et al.^36^. The sample solution (1 ml) was added to 4 ml of a 0.004% solution of DPPH in methanol. The sample absorbance was recorded at 517 nm after 30 min incubation at room temperature in the dark.

The scavenging activity of the compounds on the ABTS radical cation [ABTS,2,2′-azino-bis(3-ethylbenzothiazoline)-6-sulphonic acid] was measured according to the method of Zengin et al.^37^ with slight modification. Briefly, ABTS^+^ was produced directly by reacting a 7 mM ABTS solution with 2.45 mM potassium persulphate and allowing the mixture to incubate for 12–16 h in the dark at room temperature. Prior to initiating the assay, the ABTS solution was diluted with methanol to an absorbance of 0.700 ± 0.02 at 734 nm. The sample solution (1 ml) was added to the ABTS^+^ solution (2 ml) and mixed, and the sample absorbance was recorded at 734 nm after 30 min incubation at room temperature. The corresponding IC_50_ value, which is the effective concentration at which 50% of DPPH/ABTS radicals are scavenged, was calculated for each compound and trolox, as a reference drug.

#### 4.3.3. Reducing power tests (CUPRAC and FRAP)

The cupric ion reducing activity (CUPRAC) was determined according to the method of Zengin et al.^38^. The sample solution (0.5 ml) was added to a premixed reaction mixture containing CuCl_2_ (1 ml, 10 mM), neocuproine (1 ml, 7.5 mM), and NH_4_Ac buffer (1 ml, 1 M, pH 7.0). Similarly, a blank was prepared by adding the sample solution (0.5 ml) to a premixed reaction mixture (3 ml) without CuCl_2_. The absorbances of the sample and blank were subsequently recorded at 450 nm after 30 min incubation at room temperature. The absorbance of the blank was subtracted from that of the sample.

The FRAP (ferric reducing antioxidant power) assay was carried out as described by Zengin et al.^38^. The sample solution (0.1 ml) was added to the premixed FRAP reagent (2 ml) containing acetate buffer (0.3 M, pH 3.6), 2,4,6-tris(2-pyridyl)-*s*-triazine (10 mM) in 40 mM HCl, and ferric chloride (20 mM) in a ratio of 10/1/1 (*v*/*v*/*v*). The absorbance of the sample was subsequently recorded at 593 nm after 30 min incubation at room temperature. Both results were expressed as EC_50_ values, using trolox as a reference drug.

#### 4.3.4. Metal chelating activity on ferrous ions

Metal chelating activity on ferrous ions was evaluated by the method previously described^39^. Briefly, the sample solution (2 ml) was added to FeCl_2_ solution (0.05 ml, 2 mM). The reaction was initiated by the addition of 5 mM ferrozine (0.2 ml). Similarly, a blank was prepared by adding the sample solution (2 ml) to FeCl_2_ solution (0.05 ml, 2 mM) and water (0.2 ml) without ferrozine. Then, the sample and blank absorbance were recorded at 562 nm after incubation for 10 min at room temperature. The absorbance of the blank was subtracted from that of the sample. EDTA was used as a positive control and this property was expressed as IC_50_ value for each compound.

### 4.4. Molecular modelling

The Schrödinger suite 2018-1^40^ was used to perform all molecular modelling studies with the OPLS3^41^ as force field. Three-dimensional structures of the 4-(3-nitrophenyl)thiazol-2-ylhydrazone derivatives **3**, **4**, **13**, **37** were built by means the Maestro GUI and their oral absorption and blood–brain barrier permeation were theoretically predicted by the QikProp^42^ tool. Target protein structures were obtained from the Protein Data Bank (PDB)^43^. In particular, the PDB crystallographic entries 6FW0^44^, 2Z5X^45^, 4M0E^46^, and 1P0I^47^ were selected as theoretical models for hMAO-B, hMAO-A, hAChE, and hBuChE, respectively. Each target structure was submitted to preliminary manipulations by adding missing hydrogen atoms, deleting water molecules, removing co-crystallised ligands, and fixing FAD bonding orders. Molecular docking simulations were carried out with Glide^48^ flexible ligand implementation using the extra precision (XP) search algorithm. For the protein binding sites, a grid box of about 64,000 A3 centred on the FAD N5 atom in the MAOs and on catalytic Ser203 in the ChEs was considered. The default docking scoring function was applied for ranking ligand binding modes. Finally, ZINC PAINS Pattern Identifier^49^ was used to evaluate if our derivatives could be considered as potential pan assays interfering or aggregator compounds.

## 5. Results and discussion

### 5.1. In vitro MAO inhibition study

The synthesised compounds reported here share a similar structure, with the principal differences among compounds **1–37** occurring at the **X** and **Y** substituents as shown in Scheme 1. These substitutions play a fundamental role and affect the activity and selectivity index (SI) of inhibition of the two hMAO isoforms. SI <1 indicates selectivity for MAO-A, whereas SI >1 indicates selectivity for the MAO-B isoform. All the tested compounds were found to inhibit the hMAO enzymes with selectivity for the hMAO-B isoform (Tables 1 and 2). The presence of aromatic/heterocyclic systems as **Y** moiety was effective in order to obtain derivatives that selectively inhibit hMAO-B in the nanomolar range. Among these compounds, the most active towards this isoform were the derivatives **4** (IC_50_ hMAO-B = 0.0018 μM) and **5** (IC_50_ hMAO-B = 0.0025 μM), both containing methyl as **X** group and respectively, thiophen-2-yl and thiophen-3-yl as **Y** substituent. Comparing the inhibitory activities of compound **4** and compound **3**, which contains H as **X** group, it can be noted that there was only a modest decrease in MAO-B inhibition activity for **3**, while a reduction in inhibition activity was more noticeable for hMAO-A (IC_50_ hMAO-A = 57.2 μM). This increased the SI by approximately 10-fold (from 922 for compound **4** to 8412 for compound **3**). A similar result was obtained for compound **6** where substitution on thiophenyl ring led to a reduction in inhibition activity towards hMAO-A compared to derivative **4**. Even though hMAO-B inhibition was also slightly reduced, the SI increased significantly (compare **6** vs. **4**). Among derivatives possessing furan as **Y** substituent (**1** and **2**), only compound **1** exhibited inhibition activity in the nanomolar range for hMAO-B (IC_50_ hMAO-B = 0.095 μM). The lower activity of **2** suggests that isosteric replacement of the oxygen atom for a sulphur is unfavourable for MAO-B inhibition (compare **2** vs. **4**). Within the set of compounds containing the phenyl ring as **Y** moiety, we observed the best inhibitory activity for derivative **11** (IC_50_ hMAO-B = 0.0071 μM), which contains methyl as **X** substituent. The absence of the methyl group (**X** = H, compound **7**) or its elongation to **X** = ethyl (compound **12**) were detrimental for MAO-B inhibition activity. Nitro-substitution on the phenyl ring, as with compounds **8–10** and **13–15**, was advantageous when done in the *ortho*-position (compound **13**, IC_50_ hMAO-B = 0.0044 μM), a modification that also improved the selectivity compared with the unsubstituted derivative **11** (SI**_11_** = 666, SI**_13_** = 3477, respectively). On the other hand, substitution with an acetyl moiety in the *para-*position of phenyl ring reduced activity and selectivity as observed for compound **31** (IC_50_ hMAO-B = 42.1 μM, SI**_31_** = 5.4). Compounds substituted with pyridine as **Y** moiety (**16–21**) possess nanomolar activity towards hMAO-B (0.014 μM < IC_50_ hMAO-B < 0.212 μM), while poor potencies were recorded for hMAO-A inhibition, with IC_50_ in the micromolar range (9.78 μM < IC_50_ hMAO-A < 156 μM). Most of the compounds possessing a hetero-bicyclic system as **Y** moiety (compounds **23–27**) showed the loss of activity and selectivity, except for compound **33** that contains a coumarin moiety. Coumarin derivatives are well known to inhibit the hMAO enzymes as previously reported^50^. Naphthyl as **Y** group was effective only when substituted in the α-position (compare **28** and **29** vs. **30**), while the presence of very bulky groups such as phenanthrene (compound **34**) reduced activity towards both isoforms (IC_50_ hMAO-A = 101 μM, IC_50_ hMAO-B = 80.5 μM). Finally, we evaluated the effect of dimerisation with compound **36** and the reduction of nitro group to obtain amine functionality with compound **37**. The IC_50_ values showed that the dimerisation negatively affected inhibition activity, and this is likely related to the steric hindrance in the MAO active site. Compound **37**, on the other hand, showed loss of MAO-B inhibitory activity, which highlights the importance of the nitro group on the meta position for this scaffold (compare **37** vs. **3**). Conversely, the MAO-A inhibitory activity was not affected by nitro reduction.

**Table 1.** Inhibitory activity (IC_50_) and selectivity index (SI) of compounds **1–35** towards hMAO-A and hMAO-B.




^a^Values are the mean ± SD of triplicate determinations.

^b^Selectivity index for the MAO-B isoform, given as the ratio: (IC_50_ hMAO-A)/(IC_50_ hMAO-B).

**Table 2.** Inhibitory activity (IC_50_) and selectivity index (SI) of compounds **36** and **37** towards hMAO-A and hMAO-B.

^a^Values are the mean ± SD of triplicate determinations.

^b^Selectivity index for the MAO-B isoform, given as the ratio: (IC_50_ hMAO-A)/(IC_50_ hMAO-B).

Reversibility of MAO inhibition is an important factor to consider when evaluating the inhibition properties of potential inhibitors. Irreversible MAO inhibitors, particularly of the MAO-A isoform, are associated with dangerous adverse effects such as the cheese reaction, which occurs when irreversible MAO-A inhibitors are taken with tyramine-rich food^51^. To demonstrate that the 4-(3-nitrophenyl)thiazol-2-ylhydrazone derivatives studied here are reversible MAO inhibitors, compounds **4** and **37** were selected as test compounds. The reversibility of MAO-B inhibition was evaluated by incubating MAO-B in the presence of the test compounds (at a concentration of 4 × IC_50_) for 15 min, and subsequently dialyzing the samples for 24 h. The samples were diluted twofold and the residual MAO-B activity was measured. For reversible inhibition, dialysis is expected to reverse inhibition by the test inhibitors and restore enzyme activity to the level of the negative control. The negative control consisted of similar incubation and dialysis in the absence of inhibitor and represented 100% enzyme activity, while as positive control, these studies were carried out in the presence of the irreversible MAO-B inhibitor, (*R*)-(−)-deprenyl. A final experiment consisted of incubations containing MAO-B and tested inhibitors which were not dialyzed, but maintained for 24 h.

**Figure 1.**
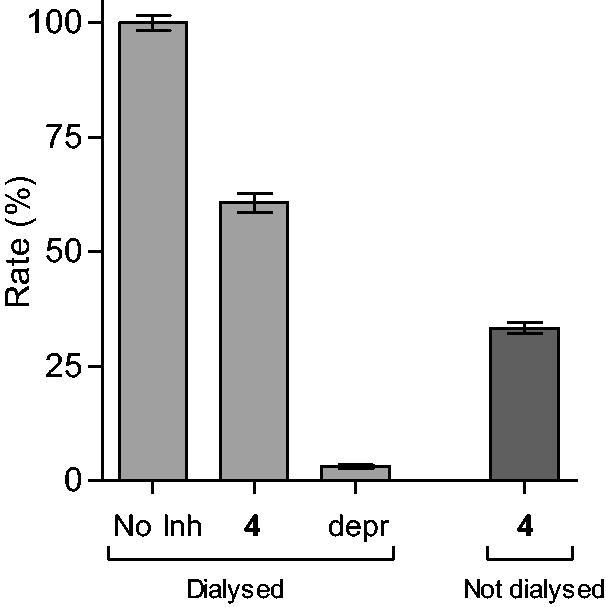
Reversibility of the inhibition of hMAO-B by **4**. hMAO-B and **4** (at 4 × IC_50_) were preincubated for 15 min, dialyzed for 24 h and the residual enzyme activity was measured (**4**, dialyzed). hMAO-B was similarly preincubated in the absence (No Inh, dialyzed) and presence of the irreversible inhibitor, (*R*)-(−)-deprenyl (depr, dialyzed), and dialyzed. For comparison, the residual hMAO activity of undialysed mixtures of hMAO-B with **4** is also shown (**4**, not dialyzed).

**Figure 2.**
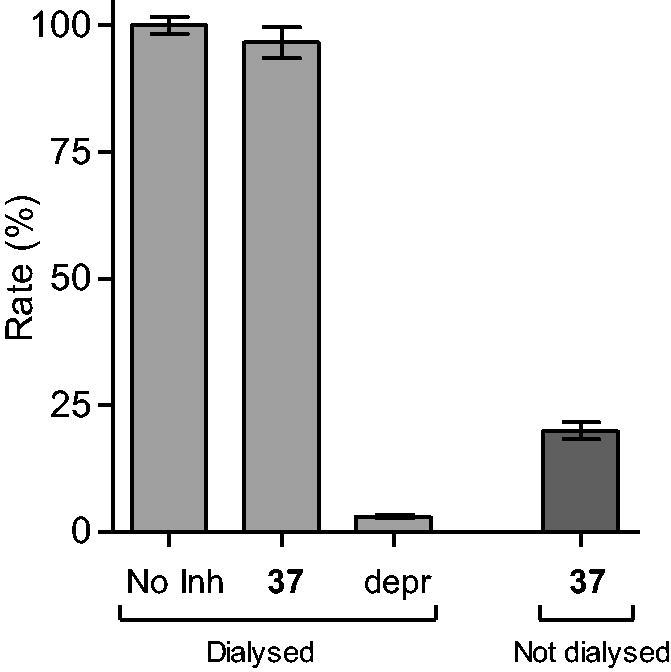
Reversibility of the inhibition of hMAO-B by **37**. hMAO-B and **37** (at 4 × IC_50_) were preincubated for 15 min, dialyzed for 24 h and the residual enzyme activity was measured (**37**, dialyzed). hMAO-B was similarly preincubated in the absence (No Inh, dialyzed) and presence of the irreversible inhibitor, (*R*)-(−)-deprenyl (depr, dialyzed), and dialyzed. For comparison, the residual hMAO activity of undialysed mixtures of hMAO-B with **37** is also shown **(37**, not dialyzed).

The results are given in Figures 1 and 2, and show that dialysis restored MAO-B activity for both **4** and **37**, with the residual activity at 61 and 97%, respectively. Compound **37** was thus a fully reversible MAO-B inhibitor, while **4** was partially reversible, which was likely due to tight-binding of this high potency inhibitor to the MAO-B active site. For (*R*)-(−)-deprenyl, dialysis did not restore enzyme activity with the residual MAO-B activity at 3% of the negative control value. In undialysed mixtures of **4** and **37**, inhibition persisted with the residual activity at 33 and 20%, respectively.

**Figure 3.**
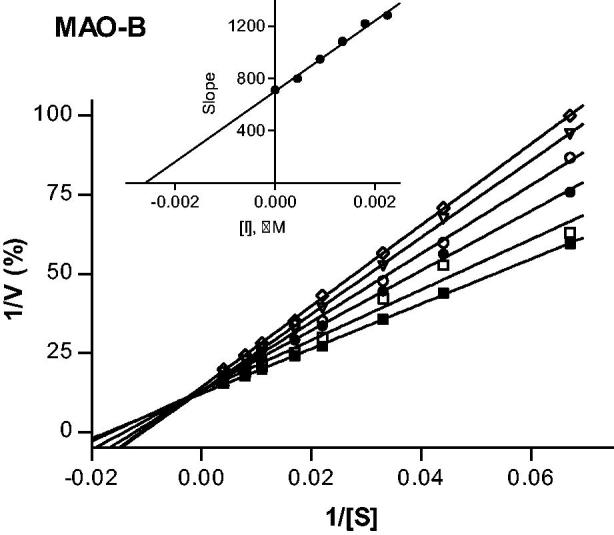
Lineweaver–Burk plots of hMAO-B activities in the absence (filled squares) and presence of various concentrations of compound **4** (IC_50_ = 0.0018 μM). For these studies the concentrations of compound **4** employed were ¼ × IC_50_, ½ × IC_50_, ¾ × IC_50_, 1 × IC_50_ and 1¼ × IC_50_. The inset is a graph of the slopes of the Lineweaver–Burk plots versus inhibitor concentration (*K*_i_ = 0.0026 μM).

**Figure 4.**
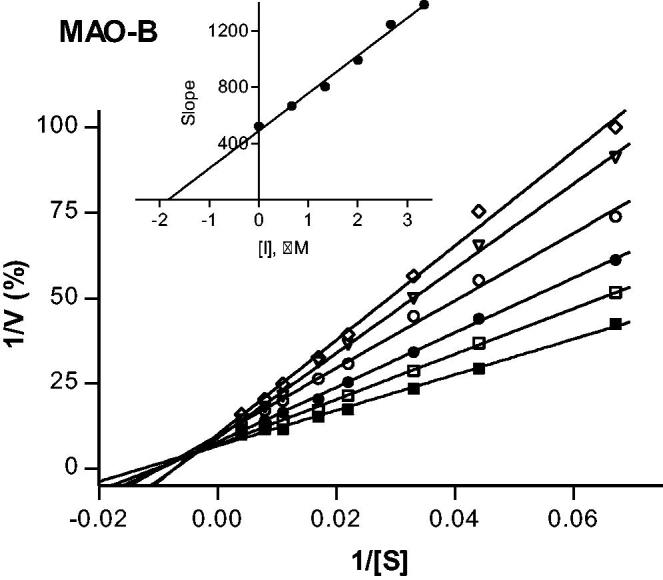
Lineweaver–Burk plots of hMAO-B activities in the absence (filled squares) and presence of various concentrations of compound **37** (IC_50_ = 2.67 μM). For these studies the concentrations of compound **37** employed were: ¼ × IC_50_, ½ × IC_50_, ¾ × IC_50_, 1 × IC_50_ and 1¼ × IC_50_. The inset is a graph of the slopes of the Lineweaver–Burk plots versus inhibitor concentration (*K*_i_ = 1.84 μM).

To provide further support for the reversibility of MAO-B inhibition by compounds **4** and **37**, a set of Lineweaver–Burk plots was constructed for each inhibitor at the following inhibitor concentrations: ¼ × IC_50_, ½ × IC_50_, ¾ × IC_50_, 1 × IC_50_, and 1¼ × IC_50_. The substrate, kynuramine, was used at 15–250 µM for each line. The results are given in Figures 3 and 4, and show that the Lineweaver–Burk plots for both inhibitors are indicative of competitive, and therefore reversible inhibition. The lines of the plots intersect on the y-axis and a replot of the slopes versus inhibitor concentration yields a linear line, from which the enzyme-inhibitor dissociation constant (*K*_i_) was estimated (*K*_i_ = −*x*-axis intercept). For compounds **4** and **37**, *K*_i_ values of 0.0026 and 1.84 µM are estimated.

### 5.2. *In vitro* antioxidant assays

It is a well-known that MAO-mediated oxidative metabolism of monoamines leads to the production of hydrogen peroxide as a by-product. Subsequently, this chemical species may be converted to free radicals through the Fenton reaction and thus contributes to the oxidative stress. Uncrontrolled increases in the concentrations of these radicals initiates oxidative damage to several cellular components. Thus, the prevention of ROS generation along with MAO inhibition is an important strategy to prevent or limit neurotoxicity in NDDs. The capability of the most promising MAO inhibitors of this study to act as antioxidant agents has been evaluated *in vitro* by five experimental approaches, using trolox as the reference compound. As it can be seen from the results in Table 3, the tested compounds showed antioxidant activity that is only slightly lower than trolox, whereas in the reducing power assay (important for the inhibition of the Fenton reaction between metal ions and the by-products of MAO-mediated reaction) and total antioxidant capacity in the phosphomolybdenum (PhosphoMo) assay, the test and reference compounds displayed almost comparable potencies. Chelating activity of ferrous ions, however, was almost absent for this scaffold. These ancillary biological activities could be useful for the treatment of multifactorial neurodegenerative diseases where oxidative stress enhances cognitive impairment and inflammatory processes.

**Table 3.** Antioxidant and chelating activities of the most promising derivatives compared to the reference drugs, EDTA and trolox.

Samples	Chelating activity	Antioxidant assays (mM)				
	IC_50_ (mM)	PhosphoMo (EC_50_)	FRAP (EC_50_)	CUPRAC (EC_50_)	DPPH (IC_50_)	ABTS (IC_50_)
**3**	>5	1.84 ± 0.18	0.41 ± 0.02	0.59 ± 0.01	1.19 ± 0.01	1.05 ± 0.01
**4**	>5	1.68 ± 0.09	0.53 ± 0.02	0.52 ± 0.02	1.17 ± 0.01	1.04 ± 0.01
**5**	>5	1.16 ± 0.03	0.52 ± 0.02	0.48 ± 0.03	1.21 ± 0.01	1.05 ± 0.02
**6**	>5	1.25 ± 0.05	0.62 ± 0.07	0.49 ± 0.01	1.18 ± 0.01	1.08 ± 0.01
**7**	>5	1.29 ± 0.16	0.66 ± 0.04	0.66 ± 0.01	1.38 ± 0.01	1.04 ± 0.01
**8**	>5	1.70 ± 0.05	0.47 ± 0.04	0.44 ± 0.02	1.47 ± 0.01	1.04 ± 0.01
**11**	>5	1.46 ± 0.08	0.59 ± 0.01	0.55 ± 0.02	1.28 ± 0.01	1.04 ± 0.01
**13**	>5	3.10 ± 0.36	0.45 ± 0.04	0.40 ± 0.01	1.59 ± 0.01	1.04 ± 0.01
**16**	>5	1.13 ± 0.01	0.51 ± 0.02	0.53 ± 0.02	1.32 ± 0.01	1.06 ± 0.02
**18**	>5	4.70 ± 0.97	0.35 ± 0.01	0.58 ± 0.01	1.37 ± 0.01	1.04 ± 0.01
**19**	>5	2.64 ± 0.34	0.38 ± 0.02	0.51 ± 0.01	1.36 ± 0.01	1.04 ± 0.01
**20**	>5	1.77 ± 0.07	0.32 ± 0.02	0.42 ± 0.02	1.29 ± 0.01	1.04 ± 0.01
**21**	>5	4.48 ± 0.18	0.27 ± 0.01	0.46 ± 0.02	1.34 ± 0.02	1.04 ± 0.01
**22**	3.86 ± 0.29	1.28 ± 0.03	0.48 ± 0.04	0.91 ± 0.02	1.34 ± 0.01	1.04 ± 0.01
**28**	>5	4.93 ± 0.83	0.41 ± 0.01	0.51 ± 0.03	1.30 ± 0.01	1.05 ± 0.01
**29**	>5	9.39 ± 0.31	0.53 ± 0.02	0.46 ± 0.01	1.42 ± 0.01	1.04 ± 0.01
**Trolox**	–	1.13 ± 0.05	0.16 ± 0.01	0.24 ± 0.02	0.40 ± 0.01	0.66 ± 0.02
**EDTA**	0.02 ± 0.001	–	–	–	–	–

**Scheme 2.**
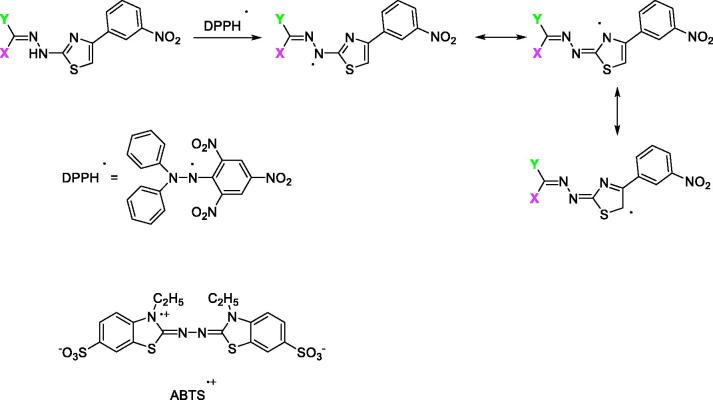
The proposed mechanism for the antioxidant ability of compounds **1–37**.

The proposed mechanism for the antioxidant activity of this scaffold of compounds is depicted in Scheme 2 using DPPH/ABTS. First the N–H group can readily donate a hydrogen radical to the DPPH/ABTS radical and generate a new radical species which is stabilised by resonance through the thiazole ring and the = C–N–N=C moiety. The new radical can be further stabilised by resonance through imine and thiazole structures. Since, the antioxidant activities using the ABTS and DPPH assay are comparable, it may be assumed that imine and thiazole moieties can participate in single electron transfer and lead to discrete antioxidant activity. Moreover, other resonance structures can be present^52^.

### 5.3. Molecular modelling

**Figure 5.**
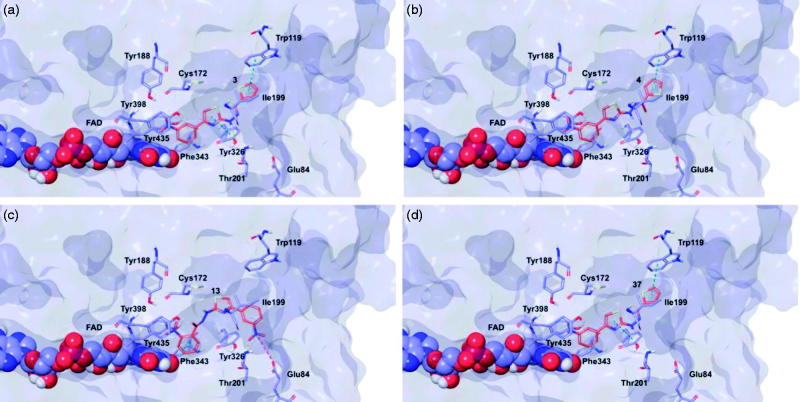
Glide top poses of compounds (a) **3**, (b) **4**, (c) **13**, and (d) **37** in the hMAO-B active site. Ligands are depicted in orange carbon polytube, the FAD is shown in lilac coloured CPK and the most relevant residues are reported in lilac carbon polytube. Hydrogen bonds, cation–π and π–π interactions are displayed in yellow, green and light blue respectively.

**Figure 6.**
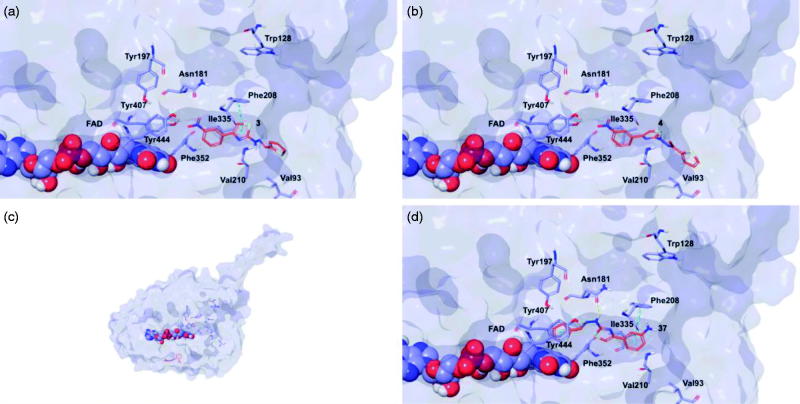
Glide top poses of compounds (a) **3**, (b) **4**, (c) **13**, and (d) **37** in the hMAO-A active site. Ligands are depicted in orange carbon polytube, the FAD is shown in lilac coloured CPK and the most relevant residues are reported in lilac carbon polytube. Hydrogen bonds, cation–π and π–π interactions are displayed in yellow, green and light blue respectively.

Target recognition of the most interesting compounds **3**, **4**, **13**, and **37**, as suggested by the experimental data, was investigated by molecular modelling simulations. In agreement with experimental data, docking results proposed, in general, preferred hMAO-B recognition compared to the A isoform. Indeed, all evaluated compounds presented better theoretical affinity for hMAO-B and thus more productive interactions with this isoform (Figure 5). For compound **13** no poses in hMAO-A active site were obtained (Figure 6).

**Figure 7.**
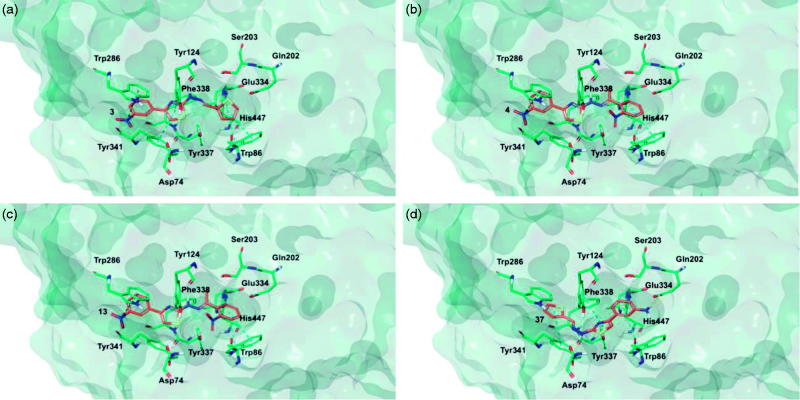
Glide top poses of compounds (a) **3**, (b) **4**, (c) **13**, and (d) **37** in the hAChE active site. Ligands are reported in orange carbon polytube and the most relevant residues are shown in green carbon polytube. Hydrogen bonds, cation–π and π–π interactions are displayed in yellow, green and light blue, respectively.

**Figure 8.**
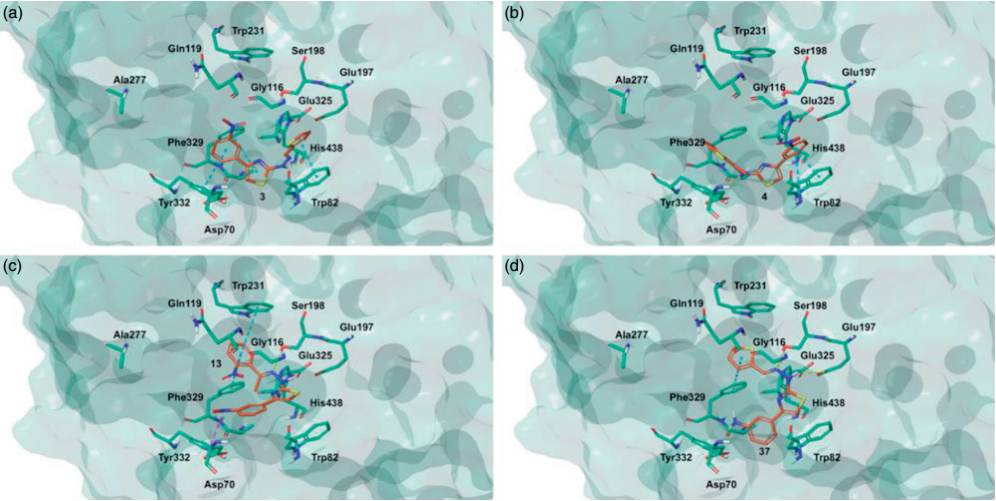
Glide top poses of compounds (a) **3**, (b) **4**, (c) **13**, and (d) **37** in the hBuChE active site. Ligands are reported in orange carbon polytube and the most relevant residues are shown in green carbon polytube. Hydrogen bonds, cation–π and π–π interactions are displayed in yellow, green and light blue, respectively.

The top poses obtained for **3**, **4**, and **37** showed similar orientations in the MAO-B active site, with the phenyl ring substituted at the meta position by nitro or amino group directed towards the FAD cofactor. A similar orientation for **13** appeared in a lower ranked pose only, since the best-scored pose showed interactions between the nitro group and the side chains of Thr201 and Glu84 located at the entry of the active site. The reduction of the nitro group did not affect the docking outcomes to a significant degree, both in term of binding geometries and affinity energy values. In fact, the most energetically favourable orientations of **3** and **37** were superimposable and both were involved in stacking interactions with Tyr326 and Trp119 by means of the thiazole ring. The binding mode of **3** was further stabilised by a hydrogen bond between the hydrazone nitrogen atom and the Tyr326 side chain resulting in a moderately better theoretical affinity. The substitution of the hydrazone hydrogen atom by a methyl group in compound **4** led to a slightly different pose, characterised by the loss of the interactions produced by the thiazolylhydrazone core. In the hMAO-A binding site, **3** and **4** were oriented with the nitro group toward the cofactor, but binding occurred towards to the gorge entry. Such poses were strongly penalised with respect to the corresponding poses in the hMAO-B active site. This observation may be due to the replacement of Tyr326 in hMAO-B by Ile335 in hMAO-A. Furthermore, unfavourable steric clashes with Val210 and Ile335 were present. Conversely to hMAO-B, the reduction of the nitro group in **37** induced an opposite binding mode in which the thiazole substituent was directed towards the FAD. Steric hindrance between **37** and Tyr407 and Tyr444 was unfavourable for ligand-target recognition. Docking of our derivatives in both ChEs (Figures 7 and 8) did not suggest a remarkable selectivity for a particular isoform. In hAChE, **3**, **4** and **13** displayed very similar binding modes and interacted with Trp286, Tyr124, Tyr341, and Trp86. Such poses seemed to be determined by the *m*-nitrophenyl moiety which interacted with the external tryptophan residue (Trp286). In fact, for **37** the opposite orientation occurred due to the loss of this productive interaction although contacts with the tyrosine residues and with the internal tryptophan (Trp86) were maintained. In hBuChE, where Ala277 replaces the hAChE Trp286, the compounds better recognised the internal Trp82 (corresponding to Trp86 in hAChE).

Computed pharmacokinetic properties indicated that all the derivatives evaluated may possess good absorption after oral administration and the CNS availability of **3**, **4**, and **37** is comparable to known CNS drugs (Table 4)^53^. None of the considered compounds were proposed as PAINS or aggregators by ZINC filter.

**Table 4.** Theoretical properties and docking scores of the most interesting compounds for binding to the hMAOs and hChEs.

Compound	%OA^a^	QlogBB^b^	Docking Score^c^			
			hMAO-A	hMAO-B	hAChE	hBuChE
**3**	95.14	−0.83	0.88	−9.86	−6.80	−6.80
**4**	100.00	−0.70	−6.10	−9.02	−7.03	−4.71
**13**	92.18	−1.17	0.81	−4.22	−5.39	−5.55
**37**	100.00	−0.47	−4.53	−9.45	−7.02	−7.04

^a^Percentage of oral absorption.

^b^Blood–brain barrier permeation as pLog(IN/OUT).

^c^XP docking score in kcal/mol of the top poses.

## 6. Conclusion

4-(3-Nitrophenyl)thiazol-2-ylhydrazones were demonstrated to be a very interesting scaffold which can be used to design high potency hMAO-B inhibitors, with IC_50_ values in the nanomolar range. The presence of specific moieties at the hydrazone linker gave the most selective inhibition of this isozyme. These compounds are also characterised by a reversible and competitive mode of hMAO-B inhibition, as well as by discrete antioxidant properties. Moreover, the physical-chemical properties of the designed molecules were predicted *in silico* and were found to fit the requirements for CNS penetration. Additional interaction with the cholinesterases (AChE and BuChE) may be possible which suggests that 4-(3-nitrophenyl)thiazol-2-ylhydrazones are novel multi-target compounds and potential leads for the design of future therapies for NDDs.
